# Alloplastic total temporomandibular joint (TMJ) replacement registry: a protocol for a prospective global multicentre observational cohort study

**DOI:** 10.1136/bmjopen-2025-113558

**Published:** 2026-03-09

**Authors:** Andreas Thor, Martin Bengtsson, Krzysztof Dowgierd, Sergey Epifanov, Andreas M Fichter, Drago Jelovac, Philippe N Korn, Johanna L Nilsson, Gabriel Pastore, Vivesh Rughubar, Wenko Smolka, Jani Talvilahti, Daniel Thiem, Florian Thieringer, Mattias Ulmner, Eppo B Wolvius, Ignacio Zubillaga Rodríguez, Vinay V Kumar

**Affiliations:** 1 Plastic and Oral and Maxillofacial Surgery, Uppsala universitet, Uppsala, Sweden; 2 Department of Oral and Maxillofacial Surgery, Skånes universitetssjukhus, Lund, Lund, Skåne County, Sweden; 3 Wojewódzki Szpital Rehabilitacyjny dla Dzieci w Ameryce, Olsztynek, Warmian-Masurian Voivodeship, Poland; 4 Pirogov Russian National Research Medical University, Moscow, Russian Federation; 5 University Hospital Leipzig, Clinic and Polyclinic for Oral and Maxillofacial Surgery, Leipzig, Saxony, Germany; 6 Clinic for Maxillofacial Surgery, University of Belgrade Faculty of Medicine, Belgrade, Serbia; 7 Hannover Medical School, Hannover, Lower Saxony, Germany; 8 Department of Oral and Maxillofacial Surgery, Rigshospitalet, Copenhagen, Capital Region of Denmark, Denmark; 9 Beneficência Portuguesa de São Paulo, São Paulo, Brazil; 10 Department of Oral and Maxillofacial Surgery, King Edward VIII Hospital, Durban, KwaZulu-Natal, South Africa; 11 Department of Oral and Maxillofacial Surgery and Facial Plastic Surgery, LMU Hospital, Musculoskeletal University Center Munich, Munich, Bavaria, Germany; 12 Department of Oral and Maxillofacial Surgery, Falun Hospital, Falun, Dalarna County, Sweden; 13 Department of Oral and Maxillofacial Surgery, University Medical Center, Johannes Gutenberg University Mainz, Mainz, Rhineland-Palatinate, Germany; 14 Oral and Cranio-Maxillofacial Surgery, Universitätsspital Basel, Basel, Basel-Stadt, Switzerland; 15 Karolinska University Hospital, Stockholm, Stockholm County, Sweden; 16 Department of Oral and Maxillofacial Surgery, Erasmus MC University Medical Center Rotterdam, Rotterdam, The Netherlands; 17 Hospital Universitario 12 de Octubre, Madrid, Community of Madrid, Spain; 18 Oral Rehabilitation Center, Bengaluru, Karnataka, India

**Keywords:** Prospective studies, REGISTRIES, ORAL & MAXILLOFACIAL SURGERY, Facial plastic & reconstructive surgery, Observational Study, Head & neck surgery

## Abstract

**ABSTRACT:**

**Introduction:**

Total alloplastic replacement of the temporomandibular joint (TMJ) is a viable treatment option for severe TMJ disorders (TMDs) unresponsive to conservative approaches, as well as for reconstruction of congenital or acquired TMJ defects. However, clinical data on indications, outcomes, complications and long-term effects remain limited, and no global registry currently exists. This study aims to address this gap by establishing an international registry to collect data from patients undergoing total alloplastic TMJ replacement systematically. The registry will document clinical indications and disease progression, explore relationships between treatments, outcomes and quality of life, identify predictors of favourable outcomes and inform future research.

**Methods and analysis:**

This international, prospective, multicentre, observational registry will enrol approximately 200 patients with TMD requiring total alloplastic TMJ replacement, with follow-up lasting up to 5 years postoperatively. The data collected will include underlying disease, treatment details, functional outcomes, patient-reported outcomes and procedure-related adverse events. The registry will also monitor patients who decline surgery and record their reasons. All treatments will adhere to the standard of care at each participating centre.

**Ethics and dissemination:**

Ethics approval was obtained from the responsible ethics committee (EC) at each participating site prior to TMJ surgery. All patients will be enrolled following an informed consent process approved by the relevant EC. Study results will be disseminated through peer-reviewed publications.

Approving ECs include: Krishnadevaraya College of Dental Sciences and Hospital EC, KCDS/Ethical Comm/54/2022–23; Ethikkommission Nordwest- und Zentralschweiz, 2019–02387; University of Belgrade School of Dental Medicine EC, 36/19; National Videnskabsetisk Komité, 2401881; University of KwaZulu-Natal Biomedical Research EC, BREC/00001592/2020; Etikprövningsmyndigheten, 2019–04477; Ethikkommission Medizinische Hochschule Hannover, 8660_BO_K_2019; Ethik-Kommission an der Medizinischen Fakultät der Universität Leipzig, 080/21-lk; Comité de Ética de la Investigación con Medicamentos Hospital Universitario 12 de Octubre, 19/392; Landesärztekammer Rheinland-Pfalz EC, 2025–18012-andere Forschung/nachberatend; Local Ethical Committee at National Medical and Surgical Centre named after NI Pirogoov, LEC meeting 5; Ethikkommission bei der LMU München, 19–589; Komisja Bioetyczna przy Warmińsko-Mazurskiej Izbie Lekarskiej w Olsztynie, 12/2021; De Medisch Ethische Toetsings Commissie Erasmus MC, MEC-2019–0696 and Comissão Nacional de Ética em Pesquisa, 3.825.711.

**Trial registration number:**

NCT03991728.

**STRENGTHS AND LIMITATIONS OF THIS STUDY**

- This is the first prospective, multicentre international registry for total alloplastic temporomandibular joint (TMJ) replacement with up to 5 years of follow-up (FU).
- The registry will collect high-quality, prospective data on treatment and outcomes from a large cohort of patients undergoing alloplastic total TMJ replacement, providing broader insights into the underlying disease, treatments and outcomes.
- A limitation of the study is its observational study design, which precludes direct comparison of treatment outcomes across interventions.
- The extended FU period (60 months) may result in missing data over time.
- Differences in treatment protocols and reporting standards across participating centres may affect the consistency, quality and comparability of the collected data.

## Introduction

Temporomandibular joint (TMJ) disorders (TMDs) are a broad group of conditions affecting the TMJ, masticatory muscles and surrounding structures, leading to pain and dysfunction of the jaw and the muscles responsible for jaw movement.^1^ Patients with TMDs often present with symptoms, such as limited mouth opening, facial pain, localised facial swelling, malocclusion and facial disharmony, all of which can be severely debilitating and significantly impair daily life.^2^

TMDs affect individuals of all ages, with peak incidence from adolescence to 60 years, and are more common in women.^3 4^ Prevalence estimates range widely, from 5%–15% in earlier studies^5^,^8^ to 30%–50% in more recent reviews.^34 9^,^11^ This discrepancy likely reflects the complex, multifactorial nature of TMDs and the challenges associated with their diagnosis and classification. In addition, advances in diagnostic methods and the use of broader inclusion criteria have likely contributed to higher prevalence estimates in recent years.^3 12^

The first-line treatment for TMDs is conservative, with approaches including patient education, physical therapy, pharmacologic management, occlusal appliances and biofeedback in cases of chronic conditions, often within an interdisciplinary approach addressing physical and psychological factors.^213^,^17^ However, end-stage conditions—such as post-tumour resections, ankylosis, osteoarthritis or congenital and developmental anomalies—may severely impair mandibular function, requiring total joint replacement with alloplastic components for the condylar head and temporal fossa.^18^,^21^ The primary goals of TMJ replacement are pain relief, improved jaw function, corrected occlusion and facial deformities and improved quality of life (QoL).^2 5 14 15 22^

Although it is one of the most frequently used joints in the body, the TMJ is replaced with alloplastic implants much less frequently than joints such as the hip or knee.^23^ This may be partly attributable to the poor performance of early devices, which were associated with high failure rates, frequent revisions, suboptimal trial design and limited outcome monitoring.^7 20^ In addition, the readability, reliability, content and overall quality of online information on TMDs vary widely, which may further contribute to patients’ reluctance to pursue surgical treatment.^24^ Advances in personalised implant design, materials and biomechanics have made modern TMJ replacements safer and more effective.^1920 25^,^27^ As a result, the use of this treatment has increased, with procedure numbers rising and further growth expected in the coming years.^21^

Despite these advances, high-quality clinical data remain limited.^23 28^ Most published studies—excluding case reports—consist of retrospective series from a few centres, often with limited sample size and a potential risk of conflict of interest.^2229^,^31^ Although reported outcomes are generally positive, key questions regarding patient selection, optimal timing of intervention and whether early replacement is beneficial or should be reserved for specific patient subgroups remain unanswered. The UK has established a national TMJ replacement database,^32^ developed under the auspices of the British Association of TMJ Surgeons, to collect longitudinal outcome data within the UK. This database enables surgeons to compare outcomes with peers and share long-term results. However, no global registry currently exists. This highlights the need for international, multicentre studies to better define indications, outcomes, costs, complications and long-term effects of alloplastic TMJ replacement.

This prospective, multicentre observational registry aims to fill this gap. Beyond increasing data availability, the registry will serve as a structured tool to phenotype patients undergoing alloplastic TMJ replacement by documenting underlying disease, indications, comorbidities and broader clinical context. It will capture real-world variability in surgical techniques, implant selection, perioperative management and rehabilitation strategies across centres. In addition, the registry will capture functional outcomes, patient-reported outcomes (PROs), anticipated and procedure-related adverse events (AEs) and data on patients who decline surgery, allowing exploration of decision-making processes and barriers to treatment.

Collectively, these data will support hypothesis generation, enable stratified analyses and inform future comparative effectiveness research and registry-based trials in this field.

## Methods and analysis

### Study design and setting

This is a prospective, multicentre observational cohort study serving as a registry of patients with severe TMD from any cause, either congenital or acquired, requiring alloplastic total TMJ replacement. Table 1 summarises the sites that are currently included in the study.

**Table 1:** Details of participating sites

Participating site	Country	Region
Oral Rehabilitation Centre, Bangalore	India	Asia
University Hospital Basel, Basel	Switzerland	Europe
Clinic for Maxillofacial Surgery, University of Belgrade, Belgrade	Serbia	Europe
Rigshospitalet, Copenhagen	Denmark	Europe
King Edward VIII Hospital, Durban	South Africa	Africa
Falu Hospital, Falun	Sweden	Europe
University Hospital Leipzig, Leipzig	Germany	Europe
Skåne University Hospital, Lund	Sweden	Europe
12 de Octubre University Hospital, Madrid	Spain	Europe
Medizinische Hochschule Hannover, Hannover	Germany	Europe
Pirogov National Medical and Surgical Center (NMSC), Moscow	Russia	Europe
Ludwig Maximilian University Hospital, Munich	Germany	Europe
Wojewódzki Specjalistyczny Szpital Dziecięcy, Olsztyn	Poland	Europe
Erasmus University Medical Center, Rotterdam	The Netherlands	Europe
Karolinska University Hospital, Stockholm	Sweden	Europe
A Beneficência Portuguesa de São Paulo, São Paulo	Brazil	South America
Uppsala University Hospital, Upphsala	Sweden	Europe
Universitätsmedizin Mainz, Mainz	Germany	Europe

### Objectives

The study seeks to broaden understanding of TMDs, treatment patterns and patient outcomes, with a focus on clinical indications, regional variations and patient-reported, clinical and functional outcomes in patients undergoing alloplastic TMJ replacement. Additionally, the study aims to identify predictive factors for favourable outcomes (such as pain reduction, range of motion, occlusal status and QoL) and understand why some patients refuse TMJ replacement.

### Study procedures

In this study, patients will receive treatment based on the local standard of care, with all intervention and postoperative care performed according to the standard procedures at participating sites, as determined by the treating surgeon. The study protocol will not influence clinical decision-making, materials or surgical/imaging techniques, except for the collection of standardised data in a customised database.

**Figure 1:**
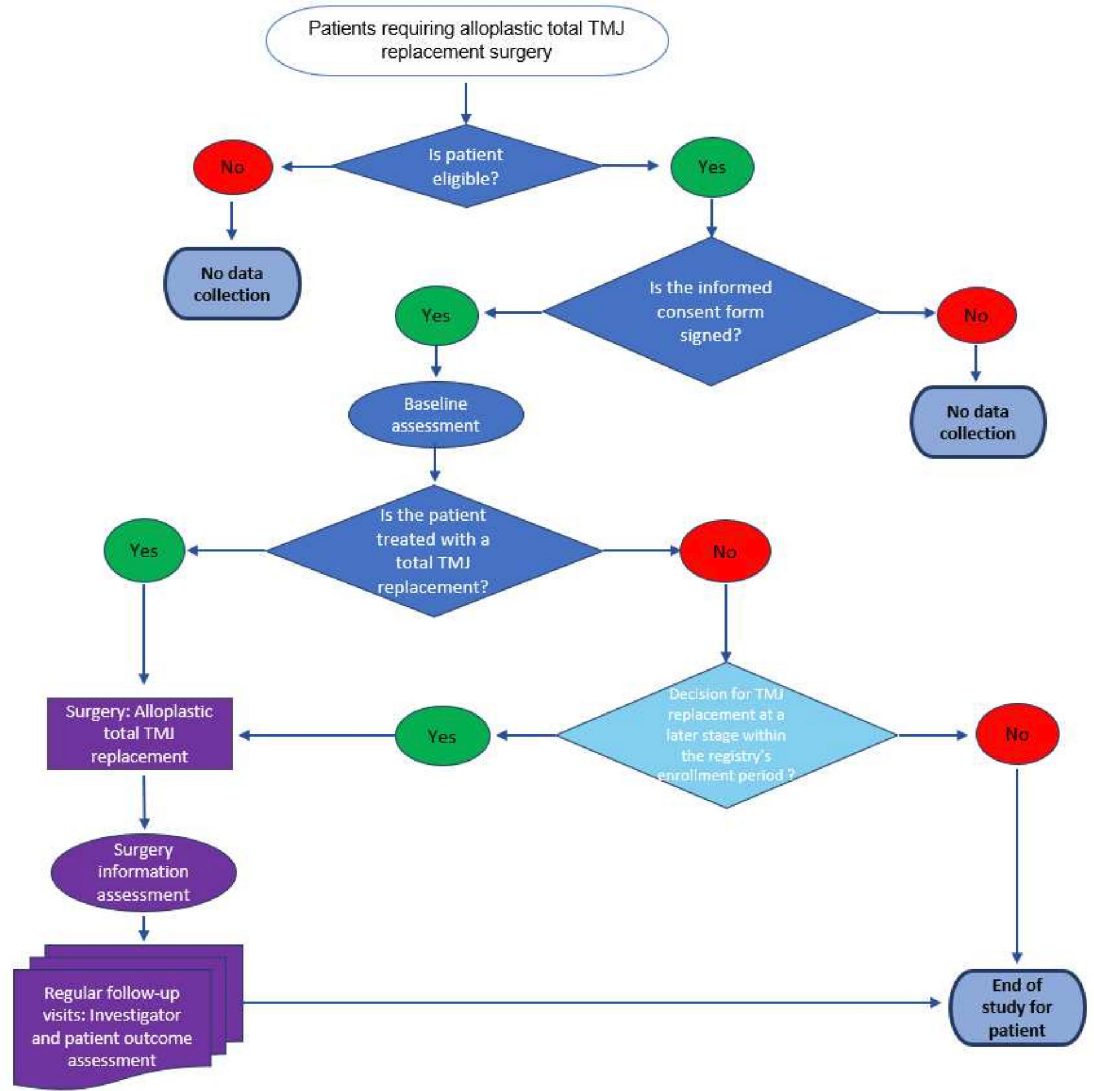
Participant workflow for the alloplastic total temporomandibular joint (TMJ) replacement registry.

### Inclusion criteria

- Aged 18 years and older.
- Patients requiring alloplastic total TMJ replacement.
- Informed consent obtained, that is:
  - Ability to understand the content of the patient information/informed consent form (ICF).
  - Willingness and ability to participate in the clinical investigation according to the research protocol.
  - Signed and dated ethics committee (EC)/institutional review board (IRB)-approved written informed consent.

### Preoperative exclusion criteria

- Patients with a recent history of substance abuse (ie, recreational drugs or alcohol) that would preclude a reliable assessment.
- Pregnant women or women planning to become pregnant within the study period.
- Participation in any other medical device or medicinal product study within the previous month that could influence the results of this study.

### Recruitment

A recruitment period of approximately 60 months is planned to enrol approximately 200 patients.

All eligible participants will be informed by a research team member about the registry’s purpose, procedures, duration, risks, benefits, alternatives to participation and data protection. Written ICF must be obtained before the TMJ replacement surgery, unless waived by the local EC/IRB or, in cases of surgery refusal, before data collection. In all cases, consent must be obtained before entering any data into the electronic case report form (eCRF).

All consented patients will be allocated a unique patient number. Each site will maintain an Identification List (IL) linking each patient’s number to their personal information. This IL is kept safe in a locked place at all times. Sites are not allowed to share the IL with any third party, except the sponsor representative, legal authorities and EC/IRB, who may have access to the IL during on-site monitoring/auditing activities.

All eligible and consented patients who underwent alloplastic total TMJ replacement will be followed up within the registry, unless their participation in the study is prematurely terminated.

Participating sites will register patients who refuse alloplastic total TMJ replacement, but these patients will not be followed up unless they choose to undergo alloplastic total TMJ replacement during the enrolment period.

### Data collection

Patients will be followed up starting approximately 10 days after treatment and then at 3, 6, 12, 24 and 60 months. A summary of the data to be collected at each visit is shown in table 2. Unscheduled visits may occur at any time during the study in case of a medical emergency or if the investigator deems it necessary for patient care. Unscheduled visits will not be documented.

**Table 2:** Study schedule

Assessment parameters	Visit 1	Visit 2	Visit 3*	Visit 4*	Visit 5*	Visit 6*	Visit 7*	Visit 8*
	Screening/preoperative	Treatment day	Postoperative 10 days (±7 days)	3 months (±30 days)	6 months (±45 days)	12 months (±60 days)	24 months (±90 days)	60 months (±90 days)
Patient information/consent	X†							
Eligibility	X†							
Baseline characteristics	X†							
Reason for refusal of TMJ replacement		X‡						
Treatment details§		X						
Pain medication	X†			X	X	X	X	X
Clinical and functional outcomes	X†		X	X	X	X	X	X
PROs	X†		X	X	X	X	X	X
Anticipated treatment or condition-related AEs	X	X	X	X	X	X	X	X
Survival			X	X	X	X	X	X
Image collection	X†		X			X	X	
Radiographical parameters¶			X			X	X	

* All postoperative FU visits within the defined time windows are calculated from the treatment day (ie, day 0).

† Assessment for patients who will undergo TMJ replacement and for patients who refused TMJ replacement.

‡ Only for patients who refused TMJ replacement.

§ For enrolled patients who underwent total TMJ replacement on one side only and have to undergo TMJ replacement on the contralateral side later during the study, details of additional surgical treatment will be collected at any time during the study.

¶ All images are taken according to local standard-of-care (routine) procedures.

AE, adverse event; FU, follow-up; PRO, patient-reported outcome; TMJ, temporomandibular joint.

#### Baseline information

Demographic data (year of birth, gender and smoking status) will be recorded for each patient. Smoking status includes whether they have a history of habitual smoking, whether they currently smoke and details on the quantity and year of use. Additionally, the use of any tobacco or nicotine products, such as cigarettes, cigars, electronic cigarettes, chewing tobacco, smokeless tobacco, nicotine patches or nicotine gum, will be documented.

Comorbidities will be assessed using the Charlson Comorbidity Index.^33^

#### Diagnosis

The disease/problem to be reported includes trauma, ankylosis, degenerative joint disease, autoimmune/inflammatory joint disease, tumours, osteonecrosis, congenital deformity, infectious diseases and others.

Signs and symptoms to be reported include the year of onset of TMJ-related problems, the affected side and the following current signs and symptoms:

- Fracture/condylar damage.
- Restricted mouth opening.
- Occlusal derangement.
- Condylar resorption/loss of height of the vertical ramus.
- Jaw movement restrictions.
- Crepitus, clicking and other joint noises.
- Failed previous implant.
- Pain.
- Difficulties in eating.
- Airway obstruction.
- Joint dislocation/subluxation.
- Other.

Previous and current treatments will be recorded, including the type of conservative treatment used (both previous and current) and the number, type and year of any prior surgical treatments. The year and reason for refusal of alloplastic total TMJ replacement will also be recorded, along with current and previous pain management treatments and oncological treatment (in cases of tumour).

For patients who refused alloplastic total TMJ replacement surgery, the reasons for refusal will be documented.

#### Treatment details

Surgical details to be documented include:

- Side (left, right or bilateral).
- Date and duration of the surgery (minutes).
- Use of preoperative virtual surgical planning.
- Implant type (stock or customised implants, brand).
- Material (mandibular and fossa components).
- Screws used (normal or back-up/safety), including side (left/right), number, length and diameter.
- Details of surgical approach as per AO standards (preauricular, retromandibular, submandibular or facelift).^34^
- Use of intermaxillary fixation (intraoperative or postoperative).
- Length of hospitalisation (days).
- Details of antibiotics and steroids.
- Details of functional rehabilitation (type and duration).
- Details of the type and frequency of pain medication (if indicated).

If applicable, additional surgical procedures (eg, dental extractions, orthognathic surgery, coronoidectomy, eminectomy, resection, reconstruction, bone or fat grafting, soft tissue flap, hardware removal) will be recorded. If a contralateral TMJ replacement is performed during the study, surgery details will be collected.

#### Premature termination of participation

Participants may be terminated early for reasons including withdrawal of consent, the investigator’s discretion (eg, non-compliance), the sponsor’s decision, loss to follow-up (FU), failure to undergo TMJ replacement (excluding those who refused surgery), death, ineligibility or other reasons. Detailed information explaining the circumstances leading to premature termination will be documented in a dropout form. For these participants, data collected up to the termination date will be censored. Censored data will be included in the analyses.

### Outcome measures

Clinical and functional outcomes, PROs and radiological parameters will be collected according to the schedule outlined in table 2.

#### Clinical and functional outcomes

Clinical and functional outcomes to be assessed include:

- Mandibular movements: measurement of interincisal/maximal opening, lateral movements and protrusive movements.^35^ Each movement is scored, and the total score is used to determine a mobility index, which distinguishes between normal mandibular mobility (0 points), slightly impaired mobility (1–4 points) and severely impaired mobility (5–20 points).
- Occlusal status: assessed according to Angle’s classification.^36^
- Dental status: recorded as edentulous, complete or partial dentition; missing teeth recorded using the Fédération Dentaire Internationale tooth numbering system.
- Mandible dysfunction: evaluated by means of the Helkimo Index (clinical dysfunction [D_i_] component),^37^ which assesses five different symptoms (impaired range of movement, impaired TMJ function, muscle pain, TMJ pain and pain on movement of the mandible). Each symptom is scored as 0 (normal), 1 (mild) or 5 (severe), giving a total score of 0–25. Patients are classified according to severity as follows:
  - D_i_ 0, clinically symptom-free (0 points).
  - D_i_ I, mild dysfunction (1–4 points).
  - D_i_ II, moderate dysfunction (5–9 points).
  - D_i_ III, severe dysfunction (10–13 points).
  - D_i_ III, severe dysfunction (15–17 points).
  - D_i_ III, severe dysfunction (20–25 points).

#### Patient-reported outcomes (PROs)

The PROs assessed in this study include the pain numeric rating scale (NRS), the jaw function NRS, the diet limitation NRS, the EuroQoL five-dimension five level (EQ-5D-5L) questionnaire and the oral health impact profile-14 (OHIP-14).

The *pain NRS* measures pain intensity on an 11-point scale from 0 (no pain) to 10 (worst pain imaginable), reflecting overall or average daily pain.

The *jaw function NRS* is also an 11-point scale from 0 (normal jaw movement) to 10 (no jaw movement), assessing subjective jaw function.

The *diet limitation NRS* evaluates diet limitation on an 11-point scale from 0 (no restrictions) to 10 (liquids only).

The *EQ-5D-5L* is a standardised instrument for assessing health-related QoL and provides a generic measure that enables comparisons across different health conditions as well as health-economic analyses. It includes five dimensions: mobility, self-care, usual activities, pain/discomfort and anxiety/depression. Each dimension has five levels of severity, ranging from no problems (level 1) to extreme problems (level 5). The level chosen by the respondent for each dimension creates a 5-digit health profile.^38^ Validated translations are available in the local languages of all participating study sites.

The *OHIP-14* was chosen to capture oral health-specific impacts that are particularly relevant to TMJ pathology and its functional consequences. The instrument measures oral health-related QoL by focusing on impairment across three functional status dimensions—social, psychological and physical—which correspond to four of the seven QoL dimensions proposed by Patrick and Bergner.^39^ All impacts measured by the OHIP are conceptualised as adverse outcomes; therefore, the instrument does not assess any positive aspects of oral health. It covers seven domains: functional limitation, physical pain, psychological discomfort, physical, psychological and social disability and handicap. Each domain includes two items, evaluated using a 5-point Likert scale from ‘never’ to ‘very often’, with higher scores indicating worse outcomes.^40^ Validated translations of the OHIP-14 are available in the local languages of all participating study sites.^41^

#### Adverse events (AEs)

Since this is an observational registry, only anticipated condition-related, treatment-related or implant-related AEs will be documented from the time of consent onwards. These include fixation failure (implant loosening, breakage or instability), dehiscence of incision, wound infections, neurological-related (facial nerve damage, Frey’s syndrome, sensory disturbances or altered hearing), bleeding, haematoma, swelling, injury to adjacent structures, ankylosis, malocclusion, sensitivity to material, heterotopic ossification, disarticulation, contralateral joint dysfunction, condylar resorption, graft loss, revision surgery and any other AEs that may affect the outcome of the registry.

These events are considered events of scientific interest, that is, events that can clearly be connected to the treatment(s) or the medical condition under investigation and will not require immediate reporting to the local EC/IRB unless they occur at a higher frequency and/or severity than that cited in the literature.

This registry can be considered a minimal-risk study, given that participation poses a very low risk compared with the standard-of-care (routine) procedures.

#### Survival

Patient’s survival will be documented at each FU visit. If a visit is missed, survival will be confirmed by phone call.

#### Covariates

Potentially important covariates (prognostic factors) to include in the analysis are:

- Age.
- Gender.
- Anxiety/depression.
- Number of previous surgeries (TMJ-related).
- Onset of disease.
- Underlying disease.
- Use of pain medications.
- Smoking.
- Type of implant.
- Geographic region.
- Baseline scores.
- Unilateral/bilateral joint replacement.

#### Radiological parameters

All images collected according to the local standard of care will be evaluated according to the following postoperative parameters:

- Displacement of the ramus component.
- Fracture of the ramus component.
- Screw loosening.
- Resorption/osteolysis of the surrounding bone.
- Status of bone grafts if used (displaced/resorbed/healed).
- Presence of ectopic bone.

All images collected per standard of care may be used for further analysis, either on-site or by a central reader. Preoperative image analysis will depend on registry findings.

### Statistical analysis

Since the objectives of this registry are descriptive and exploratory in nature, no formal hypothesis testing is planned, and all analyses will be exploratory. The planned sample size of 200 patients is based on the estimated number of eligible participants expected to be enrolled during the 2-year recruitment period. Eligible patients who refuse TMJ replacement will be included in the study but excluded from sample size calculations. Depending on the volume and quality of the data collected during the first 2 years, the registry may remain open thereafter, with patient FU of up to 5 years.

A detailed statistical analysis plan (SAP) will be finalised before the final analysis (ie, the analysis from which the results will be summarised in the final registry report). As the primary aim of the current registry is descriptive, patient characteristics, clinical data and outcomes recorded at standard-of-care scheduled FU assessments will be presented using descriptive summary statistics. Categorical variables will be summarised using frequency and percentage for each category. Continuous variables will be summarised using the mean, SD, median, IQR and minimum and maximum values. Analyses will also be stratified by clinically relevant categories, including treatment patterns.

Longitudinal clinical, functional and PRO data may be analysed using mixed-effects models for repeated measures, allowing inclusion of all available data and minimising the impact of missing data. Time-to-event analyses are planned, with survival status recorded at each scheduled FU. Kaplan-Meier methods will estimate survival probabilities, and multivariable Cox proportional hazards models will be used where event numbers permit.

Centre effects and geographic variability will be addressed using multilevel (hierarchical) models with patients nested within regions to account for clustering and differences in practice patterns. For time-to-event outcomes, region (or treating centre) will additionally be included as a covariate in Cox regression models when sample sizes permit. The AEs will be summarised at both patient and event levels, with rates calculated using the total population as the denominator, regardless of dropouts, with 95% CIs provided.

As the registry is embedded within routine clinical care, assessments are performed according to each centre’s standard practice. To support consistent data capture, all site investigators and study coordinators will receive structured training prior to enrolment, covering registry procedures, eligibility criteria, use of the eCRF and adherence to Good Clinical Practice principles. Data completeness is monitored centrally through the eCRF system, with sites supported in resolving missing or inconsistent entries.

For specific research questions, suitable patient subgroups will be identified and included. Criteria for inclusion, handling of missing data and protocol violations will be defined in the SAP before analysis.

#### Data management

Data from participants are documented in eCRF and captured in Research Electronic Data Capture. CRFs are to be completed in a timely manner and are password-protected—only authorised personnel have access. After the registry is terminated, each site will receive an electronic copy of its own data.

All images and clinical pictures taken will be de-identified and sent to the sponsor digitally.

Due to the registry’s observational nature, no data safety monitoring board or safety officer will be established.

### Current study status

To date, 18 ethically approved centres, the majority of which are European, are participating in this study (table 1). The first patient was enrolled in June 2021. The enrolment start date for each site is provided in the online supplemental table.

## Ethics and dissemination

Ethics approval was obtained from the local EC/IRB before patient enrolment. The registry has been designed and implemented in accordance with current international standards, including the International Council for Harmonisation of Technical Requirements for Pharmaceuticals for Human Use – Good Clinical Practice (ICH GCP) guidelines and International Organization for Standardization (ISO) 14155:2020, *Clinical investigation of medical devices for human subjects — Good clinical practice*, as well as the ethical principles outlined in the Declaration of Helsinki,^42^ to ensure optimal protection of patient interests. It is intended that the results of this study shall be presented at scientific meetings and published in international peer-reviewed journals. Approving ECs are listed in the online supplemental table 1 and include Krishnadevaraya College of Dental Sciences and Hospital EC, KCDS/Ethical Comm/54/2022–23; Ethikkommission Nordwest- und Zentralschweiz, 2019–02387; University of Belgrade School of Dental Medicine EC, 36/19; National Videnskabsetisk Komité, 2401881; University of KwaZulu-Natal Biomedical Research EC, BREC/00001592/2020; Etikprövningsmyndigheten, 2019–04477; Ethikkommission Medizinische Hochschule Hannover, 8660_BO_K_2019; Ethik-Kommission an der Medizinischen Fakultät der Universität Leipzig, 080/21-lk; Comité de Ética de la Investigación con Medicamentos Hospital Universitario 12 de Octubre, 19/392; Landesärztekammer Rheinland-Pfalz EC, 2025–18012-andere Forschung/nachberatend; Local Ethical Committee at National Medical and Surgical Centre named after NI Pirogoov, LEC meeting 5; Ethikkommission bei der LMU München, 19–589; Komisja Bioetyczna przy Warmińsko-Mazurskiej Izbie Lekarskiej w Olsztynie, 12/2021; De Medisch Ethische Toetsings Commissie Erasmus MC, MEC-2019–0696 and Comissão Nacional de Ética em Pesquisa, 3.825.711.

### Patient and public involvement

This protocol was designed without patient and public involvement.

## Discussion

The number of TMJ replacement surgeries has steadily increased worldwide.^20 21^ In the USA, Onoriobe *et al*^21^ reported a 38% increase in procedures from 2005 to 2014, with demand for TMJ total joint replacement devices projected to grow by another 58% by 2030. Despite this trend, high-quality clinical data are scarce, and treatment indications, surgical methods and outcome assessments vary widely among clinicians and researchers worldwide.

Currently, no comprehensive international database exists to track outcomes or treatment-related AEs, and only one national TMJ registry has been established.^32^ The proposed TMJ registry aims to address this gap as a multicentre, prospective observational study involving international sites. It will document treatments based on current standards of care and collect real-world data across various healthcare settings. This global effort will enable comparisons of treatment practices, outcomes and complication rates, support the identification of best practices and meet the critical need for standardised data collection.

The novelty of the present study protocol lies in its aim to create a large, comprehensive dataset on alloplastic TMJ replacement. Establishing a global database will support research on long-term outcomes and improvements in QoL, ultimately guiding evidence-based decision-making and optimising patient care worldwide.

This study has some limitations. The sample size of approximately 200 patients may limit the ability to detect rare complications or perform adequately powered subgroup analyses. Variability in treatment protocols across participating centres may introduce heterogeneity and residual confounding, which could affect data consistency and comparability. In addition, the use of five PROs could be burdensome for patients, potentially lowering completion rates, and the absence of a TMD-specific QoL questionnaire, such as the Diagnostic Criteria for Temporomandibular Disorders (DC/TMD),^43^ necessitating reliance on instruments adapted from studies of edentulous populations, may limit the sensitivity of outcome assessment. The registry, however, could serve as a platform for the future integration or validation of TMJ-specific PROMs as they become available, without overburdening participants during this exploratory phase.

Despite these challenges, this registry represents a significant step forward by unifying international efforts and creating a shared platform for research and clinical improvement. Leveraging broad geographic participation, the TMJ registry will serve as the largest and most comprehensive dataset to date, enabling exploration of global practice patterns, identification of predictors of outcomes and guidance for future innovation in TMJ management.

## Supplementary material

10.1136/bmjopen-2025-113558 online supplemental file 1
