# Supplementary material for: Alloplastic total temporomandibular joint (TMJ) replacement registry: a protocol for a prospective global multicentre observational cohort study
Source: BMJ Open. 2026 Mar 9;16(3):e113558. doi: 10.1136/bmjopen-2025-113558 (PMC12983869; doi:10.1136/bmjopen-2025-113558)
Supplement: online supplemental file 1 [file bmjopen-16-3-s001.docx]

| **Center** | **EC/IRB** | **EC/IRB approval date** | **Enrollment starting date** | **Approval number** | **Email address** |
| --- | --- | --- | --- | --- | --- |
| Sri Shankara Cancer Hospital and Research Center, Bangalore, India | Krishnadevaraya College of Dental Sciences and Hospital Ethics Committee (KCDSHEC) | 24.08.2022 | not enrolling | KCDS/Ethical Comm/54/2022-23 | www.kcdsh.org |
| Universitätsspital, Basel, Switzerland | Ethikkommission Nordwest-und Zentralschweiz | 27.02.2020 | 09.03.2020 | 2019-02387 | eknz@bs.ch |
| Clinic for Maxillofacial Surgery, University of Belgrade, Belgrade, Serbia | University of Belgrade School of Dental Medicine Ethics Committee | 13.07.2021 | 16.08.2021 | 36/19 | stomfak@rcub.bg.ac.rs |
| Rigshospitalet, Copenhagen, Denmark | National Videnskabsetisk Komité (NVK) | 26.06.2024 | 07.10.2024 | 2401881 | kontakt@nvk.dk |
| King Edward VIII Hospital, Durban, South Africa | University of Kwazulu-Natal Biomedical Research Ethics Committee | 06.10.2021 | 02.12.2021 | BREC/00001592/2020 | BREC@ukzn.ac.za |
| Falu Hospital, Falun, Sweden | Etikprövningsmyndigheten Uppsala läns landsting | 04.12.2019 | 19.12.2019 | 2019-04477 | registrator@etikprovning.se |
| Hannover Medical School, Hannover, Germany | Ethikkommission Medizinische Hochschule Hannover | 10.09.2019 | 17.02.2021 | 8660_BO_K_2019 | ethikkommission@mh-hannover.de |
| University Hospital Leipzig, Leipzig, Germany | Ethik-Kommission an der Medizinischen Fakultät der Universität Leipzig | 21.07.2021 | 24.08.2021 | 080/21-lk | ethic@medizin.uni-leipzig.de |
| Skåne University Hospital, Lund, Sweden | Etikprövningsmyndigheten Uppsala läns landsting | 04.12.2019 | 05.02.2021 | 2019-04477 | registrator@etikprovning.se |
| 12 de Octubre University Hospital, Madrid, Spain | Comité de Ética de la Investigación con medicamentos del HOSPITAL UNIVERSITARIO 12 DE OCTUBRE | 10.09.2019 | 15.10.2019 | 19/392 | ceicdoc@h12o.es |
| University Medical Centre of the Johannes-Gutenberg-University, Mainz, Germany | Landesärztekammer Rheinland-Pfalz | 11.03.2025 | 17.04.2025 | 2025-18012-andere Forschung / nachberatend | ethik-kommission@laek-rlp.de |
| National Medical and Surgical Center named after N.I. Pirogoov of the Ministry of Healthcare of the Russian Federation, Moscow, Russia | Local Ethical Committee at Federal State Budgetary Institution 'National Medical and Surgical Center named after N.I. Pirogoov of the Ministry of Healthcare of the Russian Federation, Russia' | 12.09.2019 | 28.08.2021 | LEC meeting 5 | ethic@pirogov-center.ru |
| Klinikum der LMU München, München, Germany | Ethikkommission bei der LMU München | 08.10.2019 | 19.11.2019 | 19-589 |  |
| Wojewódzki Specjalistyczny Szpital Dziecięcy, Olsztyn, Poland | Komisja Bioetyczna przy Warmińsko-Mazurskiej Izbie Lekarskiej w Olsztynie | 25.03.2021 | 31.05.2021 | 12/2021 | olsztyn@hipokrates.org |
| Uppsala University Hospital, Oral and Maxillofacial surgery, Uppsala, Sweden | Etikprövningsmyndigheten Uppsala läns landsting | 04.12.2019 | 17.12.2019 | 2019-04477 | registrator@etikprovning.se |
| Erasmus Medisch Centrum, Rotterdam, Netherlands | De Medisch Ethische Toetsings Commissie Erasmus MC | 29.11.2019 | 27.05.2020 | MEC-2019-0696 | metc@erasmusmc.nl |
| Hospital BP - A Beneficência Portuguesa de São Paulo, São Paulo, Brazil | COMISSÃO NACIONAL DE ÉTICA EM PESQUISA | 09.02.2020 | 07.05.2020 | 3.825.711 | conep@saude.gov.br |
| Karolinska University Hospital, Stockholm, Sweden | Etikprövningsmyndigheten Uppsala läns landsting | 04.12.2019 | 19.12.2019 | 2019-04477 | registrator@etikprovning.se |
