# Supplementary material for: Alloplastic total temporomandibular joint (TMJ) replacement registry: a protocol for a prospective global multicentre observational cohort study
Source: BMJ Open. 2026 Mar 9;16(3):e113558. doi: 10.1136/bmjopen-2025-113558 (PMC12983869; doi:10.1136/bmjopen-2025-113558)
Supplement: Supplementary file 2 [file bmjopen-2025-113558.reviewer_comments.pdf]

PEER REVIEW HISTORY

BMJ Open publishes all reviews undertaken for accepted manuscripts. Reviewers are asked to complete a checklist review form and are provided with free text boxes to elaborate on their assessment. These free text comments are reproduced below.

ARTICLE DETAILS

Title (Provisional)

Alloplastic total temporomandibular joint (TMJ) replacement registry: protocol for a prospective global multicenter observational cohort study

Authors

Thor, Andreas; Bengtsson, Martin; Dowgierd, Krzysztof; Epifanov, Sergey; Fichter, Andreas M; Jelovac , Drago; Korn, Philippe N.; Nilsson, Johanna L.; Pastore, Gabriel; Rughubar, Vivesh; Smolka, Wenko; Talvilahti, Jani; Thiem, Daniel; Thieringer, Florian; Ulmner, Mattias; Wolvius, Eppo B; Zubillaga Rodríguez, Ignacio; Kumar, Vinay V

VERSION 1 - REVIEW

**Reviewer**	**1**
**Name**	**Wieckiewicz, Mieszko**
**Affiliation**	**Wroclaw Medical University**
**Date**	**05-Jan-2026**
**COI**	**None**

The presented protocol is very important. It needs a few corrections before publication:

1. The table 1 does not include Oral Rehabilitation Center, Bangalore - 560042, India.

2. The manuscript uses broad and heterogeneous definitions of “TMJ disorders”, without anchoring them explicitly to current international diagnostic standards.

Explicitly reference DC/TMD and clearly distinguish:

intra-articular vs extra-articular conditions,

inflammatory vs degenerative vs structural pathologies,

pain-dominant vs function-dominant phenotypes.

This is critical for comparability, stratification, and predictive modeling.

3. Conditions such as tumors, congenital deformities, ankylosis, autoimmune disease, and osteoarthritis are grouped together. These entities differ fundamentally in:

pathophysiology,

prognosis,

expected outcomes after TMJ replacement.

Please introduce mandatory etiological subgroups and prespecified subgroup analyses.

4. A total of ~200 patients over 5 years across many centers is small for a global registry. Please consider clarifying that this is a pilot or foundational registry or increase the number of participants. Maybe use the sample power and size calculation to determine the number of participants.

Include a plan for registry expansion or long-term continuation.

5. The authors want to use the Helkimo index for mandible dysfuction assessment which is a big mistake. This index is outdated and beyond current global standards. Please use the DC/TMD which is a current global standard. This is a link for the DC/TMD source article: https://pubmed.ncbi.nlm.nih.gov/24482784/

6. Clearly define primary vs secondary PROMs.

7. Surgical approaches, rehabilitation, fixation, and postoperative protocols vary by center. While this reflects real-world practice, it introduces major confounding.

Define minimum reporting standards for surgical steps. Consider classifying centers by experience/volume.

8. Clarify which analyses are exploratory vs confirmatory.

Predefine key hypotheses to avoid data dredging.

9. Please report the latest original article doi:10.17219/dmp/174598 which is crucial for this topic.

10. I suggest adding the Jaw Functional Limitation Scale 8 or 20 to the PROMs. You can find it here https://inform-iadr.com/index.php/tmd-assessmentdiagnosis/dc-tmd/

In my opinion it will be valuable.

**Reviewer**	**2**
**Name**	**Sánchez Romero, Eleuterio**
**Affiliation**	**FGCU, Rehabilitation Sciences**
**Date**	**05-Jan-2026**
**COI**	**None**

Dear Authors,

Your study protocol addresses a well-defined and clinically relevant gap in oral and maxillofacial surgery: the lack of a global, prospective registry dedicated to alloplastic total temporomandibular joint replacement. The international scope of the initiative, combined with its long follow-up horizon (up to five years) and the comprehensive capture of clinical, functional, radiological, and patient-reported outcomes, places this registry in a strong position to become a reference dataset within the field.

The manuscript is, overall, clearly written, logically structured, and methodologically coherent. Ethical considerations are robustly addressed, and the protocol aligns closely with BMJ Open's rationale for publishing study protocols—namely transparency, prevention of unnecessary duplication, and facilitation of future collaboration. Importantly, the fact that the registry is already underway reinforces both its feasibility and its practical relevance.

That said, given the inherent complexity and heterogeneity of an international observational registry, there are several areas where additional clarification would meaningfully strengthen the protocol. These points relate primarily to conceptual framing, outcome hierarchy, handling of heterogeneity, and analytical transparency. The comments below are intended to refine and strengthen the work rather than to challenge its fundamental design.

Major comments

1. Conceptual framing and rationale

The Introduction provides a solid epidemiological background and a clear historical overview of alloplastic TMJ replacement. However, the justification for establishing a registry could be more explicitly framed in conceptual terms, beyond the general notion of "limited data availability."

It would be helpful to articulate the registry more clearly as a structured tool for:

– phenotyping patients undergoing TMJ replacement (including indications, comorbidities, and broader clinical context);

– capturing real-world variability in surgical techniques, implant selection, and rehabilitation strategies; and

– generating hypotheses that could inform future comparative effectiveness studies or registry-based trials.

In addition, a brief but explicit distinction between the proposed registry and existing initiatives—such as the UK TMJ database—would further clarify the added value of this study in terms of scope, variables collected, patient-reported outcomes, and international generalisability. This would position the registry more clearly within contemporary learning health system and real-world evidence frameworks.

2. Objectives and outcomes: hierarchy and alignment

The study objectives are clearly described, but they remain intentionally broad. Given the richness of the planned data collection, the protocol would benefit from a clearer hierarchy of outcomes.

Specifically, it would strengthen interpretability to explicitly distinguish between:

– primary outcomes (for example, pain intensity, mandibular function, and quality of life at predefined time points);

– key secondary outcomes (such as complication rates, revision surgery, and radiological changes); and

– exploratory outcomes, including predictive factors, regional variation, and reasons for refusal of surgery.

Clarifying which outcomes are considered hypothesis-generating versus confirmatory is particularly important to avoid over-interpretation in future analyses derived from the registry.

3. Patient-reported outcome measures (PROMs)

The selection of patient-reported outcome measures—numeric rating scales, EQ-5D-5L, and OHIP-14—is pragmatic and appropriate for a large international registry. The absence of a TMJ-specific quality-of-life instrument is acknowledged as a limitation, but this point could be more clearly contextualised.

It would be useful to briefly justify the complementary roles of OHIP-14 (oral health–specific impact) and EQ-5D-5L (generic health utility), and to clarify whether linguistic and cultural validation of these instruments is ensured across all participating countries. In addition, the authors may wish to state explicitly that the registry could serve as a platform for the future validation or integration of TMJ-specific PROMs as they become available.

4. Heterogeneity across centres

The protocol appropriately acknowledges variability in surgical techniques, implants, and standards of care across participating centres. Given the central importance of this issue, however, it deserves more explicit methodological consideration.

Clarification would be welcome regarding how centre effects and geographic variability are expected to be handled analytically in future analyses (for example, through stratified analyses or multilevel modelling). It would also be helpful to indicate whether minimum data-completeness thresholds are required for centre participation and whether any training or harmonisation procedures are planned for key assessments, such as mandibular movement measurements.

5. Statistical analysis plan (SAP)

For a protocol manuscript, the statistical section is appropriate, but it could benefit from greater anticipatory clarity. Explicitly stating that no formal hypothesis testing is planned in

initial registry reports would help manage reader expectations. Similarly, it would be useful to clarify that inferential analyses will be considered exploratory unless prespecified in the statistical analysis plan.

Additional clarification on the planned handling of missing data beyond descriptive reporting, and on whether time-to-event analyses (for example, revision surgery) are envisaged, would further strengthen this section.

Minor comments

There are a few minor points that could be addressed during revision:

– ensure consistent terminology when referring to TMJ disorders versus TMDs;

– correct minor typographical issues (for example, duplicated severity labels within the Helkimo index categories);

– consider including a simple flow diagram illustrating patient pathways (surgery versus refusal).

Recommended Literature for the Authors

The following references are not mandatory citations, but they could strengthen the conceptual, methodological, and contextual framing of the registry. Each is justified in relation to this protocol.

1. Evaluating online information on temporomandibular disorders: readability, reliability, content and quality – a scoping review protocol

BMJ Open, 2025

DOI: 10.1136/bmjopen-2025-106870

Justification:

This protocol addresses the quality and accessibility of TMD-related information from a patient-centred perspective. Its inclusion would reinforce the patient-reported and educational dimension of the present registry, particularly relevant when documenting reasons for surgery refusal and patient decision-making processes. It also aligns methodologically with BMJ Open's emphasis on transparency in protocol reporting.

2. Usefulness of biofeedback as a potentially educational treatment of temporomandibular disorders: a systematic review and network meta-analysis

BMC Oral Health, 2025

DOI: 10.1186/s12903-025-06789-3

Justification:

This high-level synthesis contextualises conservative and educational interventions within TMD management. Citing this work could help frame TMJ replacement as part of a care

continuum, clarifying which patient profiles progress from conservative management to surgical replacement.

3. Effects of an interdisciplinary approach in the management of temporomandibular disorders: a scoping review

International Journal of Environmental Research and Public Health, 2023

DOI: 10.3390/ijerph20042777

Justification:

This review highlights the importance of multidisciplinary care models in TMD. It supports the registry's rationale for collecting broad clinical and psychosocial data, acknowledging that surgical outcomes are influenced by interdisciplinary pathways before and after joint replacement.

4. Psychological and behavioral factors involved in temporomandibular myalgia and migraine: common but differentiated profiles

International Journal of Environmental Research and Public Health, 2023

DOI: 10.3390/ijerph20021545

Justification:

This study reinforces the relevance of psychological and behavioral covariates, which are partially captured in the registry (e.g., anxiety/depression, pain medication use). It provides conceptual support for including psychosocial variables when interpreting surgical outcomes and QoL trajectories.

5. Diagnostic accuracy of a portable electromyography and electrocardiography device to measure sleep bruxism in a sleep apnea population

Clocks & Sleep, 2023

DOI: 10.3390/clockssleep5040047

Justification:

Although not directly focused on TMJ replacement, this study exemplifies objective instrumental assessment of orofacial functional phenomena. It may be cited to support future extensions of the registry toward objective functional or neuromuscular measurements.

6. Altered pain perception in a young adult with childhood trauma and suspected Riley–Day syndrome: a case report

Reports, 2025

DOI: 10.3390/reports8020080

Justification:

This case illustrates atypical pain processing and central sensitization, concepts relevant when interpreting postoperative pain outcomes and variability in patient-reported responses within the registry.

7. Fourteen-year retrospective cohort study on the impact of climatic factors on chronic musculoskeletal pain

International Journal of Rheumatic Diseases, 2025

DOI: 10.1111/1756-185x.70125

Justification:

This large cohort study underscores how contextual and environmental modifiers influence chronic pain trajectories. While not specific to TMJ surgery, it supports a broader biopsychosocial interpretation of long-term pain outcomes captured by the registry.

8. Active Conservative Interventions for Obstructive Sleep Apnea: An Umbrella Review and Meta-Meta-Analysis of Systematic Reviews

Journal: Sleep Medicine, 2025

DOI: 10.1016/j.sleep.2025.108665

Justification:

Although this umbrella review focuses on obstructive sleep apnea rather than temporomandibular joint disorders, it provides a high-level methodological example of how heterogeneous clinical populations can be systematically synthesized across multiple conservative and interventional strategies. Of particular relevance to the present registry is its emphasis on stratifying patient responses based on clinical and biological profiles rather than assuming uniform treatment effects.

This perspective closely parallels the challenges faced by a global TMJ replacement registry, where patient indications, comorbidities, psychosocial factors, and prior treatments are highly variable. Including this reference could help situate the TMJ registry within a broader precision-medicine and real-world evidence framework, reinforcing the rationale for large, prospective registries as hypothesis-generating platforms rather than purely descriptive datasets.

9. Instrumental Assessment of Sleep Bruxism: A Systematic Review and Meta-Analysis

Journal: Sleep Medicine Reviews, 2024; 74:101906

DOI: 10.1016/j.smrv.2024.101906

Justification:

This systematic review and meta-analysis provides an exemplary methodological framework for the objective, instrument-based assessment of complex biological phenomena characterized by high intra-individual variability and heterogeneous clinical expression. Its in-

depth discussion of measurement validity, signal reliability, and phenotype definition is particularly relevant to the present TMJ replacement registry.

In the context of this protocol, the paper offers valuable conceptual parallels for interpreting functional outcomes, pain-related measures, and patient-reported data collected across multiple centers with differing clinical practices. Including this reference would strengthen the methodological justification for standardized yet pragmatic outcome assessment within a real-world registry and underscore the importance of distinguishing biologically and clinically meaningful subgroups within apparently homogeneous patient populations undergoing TMJ replacement.

Overall recommendation to the authors

This is a strong, timely, and well-designed protocol with clear potential for long-term impact. The suggested revisions are primarily clarificatory rather than structural and are fully compatible with an ongoing study. Addressing them would enhance transparency, methodological rigor, and the future usability of the registry data.

Kind regards,

VERSION 1 - AUTHOR RESPONSE

	**Reviewer 1:** Dr. Mieszko Wieckiewicz, Wroclaw Medical University		
1	The table 1 does not include Oral Rehabilitation Center, Bangalore - 560042, India.	Thank you for this comment. The Oral Rehabilitation Center, Bangalore (560042), India is already included in Table 1.	No change
2	The manuscript uses broad and heterogeneous definitions of “TMJ disorders”, without anchoring them explicitly to current international diagnostic standards.<br>Explicitly reference DC/TMD and clearly distinguish:<br>intra-articular vs extra-articular conditions,<br>inflammatory vs degenerative vs structural pathologies,<br>pain-dominant vs function-dominant phenotypes.<br>This is critical for comparability, stratification, and predictive modeling.	Thank you for this important comment. We agree that the reviewer raises a valid point regarding the heterogeneity of TMJ disorder definitions and the lack of explicit anchoring to international diagnostic criteria. However, while the registry is not explicitly anchored to DC/TMD criteria, it systematically records the underlying disease or indication leading to	*Page 14, line 33 has been modified as follows:*<br>Analyses will also be stratified by clinically relevant categories, including treatment patterns.<br><br>Longitudinal clinical, functional, and PRO data may be analyzed using mixed-effects models for repeated measures, allowing inclusion of all

		TMJ replacement, and this includes trauma (panfacial fractures, isolated mandibular fracture with comminute condylar trauma, or any other fracture affecting the condyles, etc), ankylosis (bony, fibrous), degenerative joint disease (Includes osteoarthritis, arthrosis), autoimmune/inflammatory joint disease (with subtypes), tumors (with subtypes), osteonecrosis (with subtypes), congenital deformity, infectious diseases, and other relevant conditions. This approach enables comprehensive documentation of intra-articular and extra-articular pathologies, as well as inflammatory, degenerative, and structural disease processes, and supports comparability, stratification, and predictive modeling.<br><br>Please also note that as the registry is already running, changes to the assessment framework are not feasible at this stage. However, subgroup and stratified analyses are planned once data collection is complete to enable meaningful classification and comparative analyses across different disease categories and clinical phenotypes.<br><br>We have clarified this rationale and the	available data and minimizing the impact of missing data. Time-to-event analyses are planned, with survival status recorded at each scheduled FU. Kaplan–Meier methods will estimate survival probabilities, and multivariable Cox proportional hazards models will be used where event numbers permit.<br><br>Centre effects and geographic variability will be addressed using multilevel (hierarchical) models with patients nested within regions to account for clustering and differences in practice patterns. For time-to-event outcomes, region (or treating centre) will additionally be included as a covariate in Cox regression models when sample sizes permit.<br><br>*Page 17, line 20 has been modified as follows:*<br><br>the absence of a TMD–specific QoL questionnaire, such as the DC/TMD (43), necessitating reliance on instruments adapted from studies of edentulous populations, may limit the sensitivity of outcome assessment.

		planned analytic strategy in the revised manuscript. We have also added the lack of explicit anchoring to DC/TMD criteria as a limitation in the discussion section of the manuscript.	
3	Conditions such as tumors, congenital deformities, ankylosis, autoimmune disease, and osteoarthritis are grouped together. These entities differ fundamentally in:<br>pathophysiology,<br>prognosis,<br>expected outcomes after TMJ replacement.<br><u>Please introduce mandatory etiological subgroups and prespecified subgroup analyses.</u>	We agree that these conditions differ substantially in pathophysiology, prognosis, and expected outcomes following TMJ replacement. Given the rarity of total alloplastic TMJ replacement, the registry was designed to be inclusive to maximise recruitment. Etiological categories are being prospectively recorded, and subgroup analyses are planned following completion of data collection, contingent on sufficient case numbers within each entity to allow meaningful analysis. We have clarified this approach in the revised manuscript.	*Page 14, line 33 has been modified as follows:*<br>Analyses will also be stratified by clinically relevant categories, including treatment patterns. Longitudinal clinical, functional, and PRO data may be analyzed using mixed-effects models for repeated measures, allowing inclusion of all available data and minimizing the impact of missing data. Time-to-event analyses are planned, with survival status recorded at each scheduled FU. Kaplan–Meier methods will estimate survival probabilities, and multivariable Cox proportional hazards models will be used where event numbers permit.<br><br>Center effects and geographic variability will be addressed using multilevel (hierarchical) models with patients nested within regions to account for clustering and differences in practice patterns. For

			time-to-event outcomes, region (or treating center) will additionally be included as a covariate in Cox regression models when sample sizes permit.
4	A total of ~200 patients over 5 years across many centers is small for a global registry. Please consider clarifying that this is a pilot or foundational registry or increase the number of participants. Maybe use the sample power and size calculation to determine the number of participants.<br>Include a plan for registry expansion or long-term continuation.	Thank you for this comment. The planned sample size should be interpreted in the context of an international prospective registry rather than a hypothesis-driven clinical trial. This registry is exploratory in nature and includes multiple outcomes with differing statistical structures, including time-to-event outcomes (i.e. FU visits will be scheduled and performed according to the local standard of care and at each scheduled visit the patient's survival will be documented), repeatedly measured continuous variables (e.g. pain NRS) and categorical outcomes (e.g. Occlusal status). As these endpoint types requires different assumptions and statistical methods, a formal power-based sample size determination is not appropriate, unless a single primary endpoint is specified, which would be inconsistent with the exploratory purpose of a registry.	*Page 14, line 19 has been modified as follows:*<br>Since the objectives of this registry are descriptive and exploratory in nature, no formal hypothesis testing is planned, and all analyses will be exploratory. The planned sample size of 200 patients is based on the estimated number of eligible participants expected to be enrolled during the 2-year recruitment period. Eligible patients who refuse TMJ replacement will be included in the study but excluded from sample size calculations. Depending on the volume and quality of the data collected during the first 2 years, the registry may remain open thereafter, with patient FU of up to 5 years.

		The target sample size of approximately 200 patients is therefore based on realistic recruitment considerations. Recruitment for total alloplastic TMJ replacement has proven challenging due to the rarity of the procedure and practical barriers to prospective enrolment, and enrolment rates have been lower than initially anticipated at most participating centres. Nonetheless, a cohort of this size represents a substantial sample in this field.<br><br>Patients will be followed for up to 5 years, and the registry may remain open beyond this period depending on recruitment volume and data quality. These points have been clarified in the revised manuscript.	
5	The authors want to use the Helkimo index for mandible dysfuction assessment which is a big mistake. This index is outdated and beyond current global standards. Please use the DC/TMD which is a current global standard. This is a link for the DC/TMD source article: https://pubmed.ncbi.nlm.nih.gov/24482784/	Thank you for this comment. We acknowledge that the DC/TMD is the current gold standard for the assessment of temporomandibular disorders, particularly internal derangement. However, this study includes patients with both articular and non-articular indications for total alloplastic TMJ replacement, such as malignancies, benign pathologies, and post-traumatic ankylosis. To	*Page 17, line 19 has been modified as follows:*<br><br>In addition, the use of five PROMs could be burdensome for patients, potentially lowering completion rates, and the absence of a TMD–specific QoL questionnaire, such as the DC/T (42), necessitating reliance on instruments adapted from studies of edentulous

		allow inclusion and assessment across this heterogeneous patient population and to enhance generalisability, the Helkimo Index was selected.<br><br>As the registry is already running, changes to the assessment framework are not feasible at this stage. We have now explicitly acknowledged this choice and its implications as a limitation of the study in the revised manuscript.	populations, may limit the sensitivity of outcome assessment.
6	Clearly define primary vs secondary PROMs.	We thank the reviewer for this comment. As this is an international, multicenter, prospective registry - i.e. a patient registry that is not a hypothesis-driven clinical study - no primary or secondary PROMS are defined. Instead, a comprehensive set of patient-reported outcome measures to characterise outcomes following total alloplastic TMJ replacement are collected. Where sample size and data completeness permit, focused subanalyses of specific PROMs will be conducted. This has been clarified in the revised manuscript.	*Page 14, line 19 has been modified as follows:*<br><br>Since the objectives of this registry are descriptive and exploratory in nature, no formal hypothesis testing is planned, and all analyses will be exploratory.
7	Surgical approaches, rehabilitation, fixation, and postoperative protocols vary by center. While this reflects real-world practice, it introduces major confounding.<br>Define minimum reporting standards for	Please see also our response to Reviewer 2, point 4. Thank you for this important comment. We agree that variation in surgical technique and	*Page 15, line 15 has been modified as follows:*<br><br>As the registry is embedded within

	surgical steps. Consider classifying centers by experience/volume.	postoperative management across centres may introduce confounding, while also reflecting real-world practice.<br><br>As this is an international prospective registry embedded within routine clinical care, surgeons are not instructed to follow standardized protocols, in order to capture contemporary practice patterns. All participating centres are of high expertise and we assume the centres will report with adequate standards for surgical steps. Any inadequacies will be detected at data analysis stage.<br><br>Key procedural details and postoperative management variables are systematically recorded in the registry to enable stratified and multivariable analyses to explore and adjust for centre-level and technique-related differences. We have clarified this approach in the revised manuscript.<br><br>In addition, we agree that centre experience may influence outcomes. We will consider stratified or sensitivity analyses based on centre volume where feasible.	routine clinical care, assessments are performed according to each centre's standard practice. To support consistent data capture, all site investigators and study coordinators receive structured training prior to enrollment, covering registry procedures, eligibility criteria, use of the electronic case report form (eCRF), and adherence to Good Clinical Practice principles. Data completeness is monitored centrally through the eCRF system, with sites supported in resolving missing or inconsistent entries. For specific research questions, suitable patient subgroups will be identified and included. Criteria for inclusion, handling of missing data, and protocol violations will be defined in the SAP before analysis.<br><br>*Page 17, line 17 has been modified as follows:*<br><br>Variability in treatment protocols across participating centers may introduce heterogeneity and residual confounding, which could affect data consistency and comparability.

		We have acknowledged residual confounding as a limitation of this study.	
8	Clarify which analyses are exploratory vs confirmatory.<br>Predefine key hypotheses to avoid data dredging.	Thank you for this comment. Please see also reply to question number 4.<br><br>We would like to clarify that this study is designed as an international, multicenter, prospective patient registry rather than a hypothesis-driven clinical study. As such, no confirmatory hypotheses are prespecified. All analyses will be exploratory in nature. The data will first be summarized using simple descriptive statistics to characterize patient demographics, treatments, and outcomes. Depending on the volume, and quality of the collected data, exploratory analyses may be conducted to investigate relationships between the different treatment options and the outcomes. We have revised the statistical analysis part of the manuscript to clarify this point	*Page 14, line 19 has been modified as follows:*<br><br>Since the objectives of this registry are descriptive and exploratory in nature, no formal hypothesis testing is planned, and all analyses will be exploratory.<br><br>*Page 14, line 33 has been modified as follows:*<br><br>Analyses will also be stratified by clinically relevant categories, including treatment patterns.<br>Longitudinal clinical, functional, and PRO data may be analyzed using mixed-effects models for repeated measures, allowing inclusion of all available data and minimizing the impact of missing data. Time-to-event analyses are planned, with survival status recorded at each scheduled FU. Kaplan–Meier methods will estimate survival probabilities, and multivariable Cox proportional hazards models will be used where event numbers permit.<br>Center effects and geographic variability

			will be addressed using multilevel (hierarchical) models with patients nested within regions to account for clustering and differences in practice patterns. For time-to-event outcomes, region (or treating center) will additionally be included as a covariate in Cox regression models when sample sizes permit.
9	Please report the latest original article doi:10.17219/dmp/174598 which is crucial for this topic.	We thank the reviewer for this suggestion and have now cited and reported the study as recommended.	See Ref. N 31
10	I suggest adding the Jaw Functional Limitation Scale 8 or 20 to the PROMs. You can find it here https://inform-iadr.com/index.php/tmd-assessmentdiagnosis/dc-tmd/<br>In my opinion it will be valuable.	Thank you for this valuable suggestion. We agree that the Jaw Functional Limitation Scale (JFLS-8 or JFLS-20) is a well-validated and informative PROM for assessing functional limitations in patients with temporomandibular disorders.<br><br>However, at this stage we are unable to add additional PROMs. The registry is already ongoing, and the selection of outcome measures was finalised at study initiation. The PROMs currently included were deliberately chosen to balance clinical	No change

		relevance with feasibility and participant burden and are considered sufficient for the exploratory aims of this study.<br><br>While many validated PROMs exist, including all of them would result in excessive data collection and reduced data quality. We believe the current set of PROMs provides a comprehensive assessment of outcomes relevant to total alloplastic TMJ replacement.	
	**Reviewer 2:** Dr. Eleuterio Sánchez Romero, FGCU		
1	Conceptual framing and rationale<br><br>The Introduction provides a solid epidemiological background and a clear historical overview of alloplastic TMJ replacement. However, the justification for establishing a registry could be more explicitly framed in conceptual terms, beyond the general notion of "limited data availability."<br><br>It would be helpful to articulate the registry more clearly as a structured tool for:<br>– phenotyping patients undergoing TMJ replacement (including indications, comorbidities, and broader clinical context);<br>– capturing real-world variability in surgical techniques, implant selection, and rehabilitation strategies; and<br>– generating hypotheses that could inform future comparative effectiveness studies or registry-based trials.<br><br>In addition, <u>a brief but explicit distinction</u>	Thank you for this suggestion. We agree and have revised the Introduction to more clearly articulate the conceptual rationale for the registry. Additionally, a brief distinction between our international registry and existing initiatives such as the UK TMJ database has now been included in the revised manuscript to clarify the added value of this study.<br><br>We hope this distinction clarifies the unique contribution of our registry and have added corresponding text to position the study more clearly within contemporary	*Page 5, line 32 has been modified as follows:*<br><br>This prospective, multicenter observational registry aims to fill this gap. Beyond increasing data availability, the registry will serve as a structured tool to phenotype patients undergoing alloplastic TMJ replacement by documenting underlying disease, indications, comorbidities, and broader clinical context. It will capture real-world variability in surgical techniques, implant selection, peri-operative management, and

	between the proposed registry and existing initiatives—such as the UK TMJ database—would further clarify the added value of this study in terms of scope, variables collected, patient-reported outcomes, and international generalisability. This would position the registry more clearly within contemporary learning health system and real-world evidence frameworks.	international research efforts.	rehabilitation strategies across centres. In addition, the registry will collect functional outcomes, patient-reported outcomes (PROs), anticipated and procedure-related adverse events, and will record patients who decline surgery, allowing exploration of decision-making processes and barriers to treatment.<br><br>Collectively, these data will support hypothesis generation, enable stratified analyses, and inform future comparative effectiveness studies and registry-based trials in this field<br><br>*Page 5, line 26 has been modified as follows:*<br><br>The UK has established a national TMJ replacement database (32) developed under the auspices of the British Association of TMJ Surgeons (BATS) to collect longitudinal outcome data within the UK. This database enables surgeons to compare outcomes with their peers and share long-term results. However, no global registry currently exists. This highlights the need

			for international, multicentre studies to better define indications, outcomes, costs, complications, and long-term effects of alloplastic TMJ replacement.
2	Objectives and outcomes: hierarchy and alignment<br><br>The study objectives are clearly described, but they remain intentionally broad. Given the richness of the planned data collection, the protocol would benefit from a clearer hierarchy of outcomes.<br><br>Specifically, it would strengthen interpretability to explicitly distinguish between:<br>– primary outcomes (for example, pain intensity, mandibular function, and quality of life at predefined time points);<br>– key secondary outcomes (such as complication rates, revision surgery, and radiological changes); and<br>– exploratory outcomes, including predictive factors, regional variation, and reasons for refusal of surgery.<br><br>Clarifying which outcomes are considered hypothesis-generating versus confirmatory is particularly important to avoid over-interpretation in future analyses derived from the registry.	Thank you for this comment. Please see also our reply to Reviewer 1, question 4, 6, and 8.<br><br>We would like to clarify that this study is designed as an international, multicenter, prospective patient registry rather than a hypothesis-driven clinical study. As such, no formal primary or secondary outcomes are predefined. Instead, the registry is intentionally broad and collects a comprehensive set of clinical, functional, and patient-reported measures to reflect real-world practice.<br><br>Therefore, all outcomes collected within the registry are considered hypothesis-generating. Any confirmatory analyses would only be undertaken in future studies with prespecified hypotheses and appropriate statistical planning. We have clarified this explicitly in the revised manuscript to avoid over-	*Page 14, line 19 has been modified as follows:*<br><br>Since the objectives of this registry are descriptive and exploratory in nature, no formal hypothesis testing is planned, and all analyses will be exploratory.<br><br>*Page 14, line 33:* Analyses will also be stratified by clinically relevant categories, including treatment patterns. Longitudinal clinical, functional, and PRO data may be analyzed using mixed-effects models for repeated measures, allowing inclusion of all available data and minimizing the impact of missing data. Time-to-event analyses are planned, with survival status recorded at each scheduled FU. Kaplan–Meier methods will estimate survival probabilities, and multivariable Cox proportional hazards models will be used

		interpretation of registry findings.	where event numbers permit.
3	Patient-reported outcome measures (PROMs)<br><br>The selection of patient-reported outcome measures—numeric rating scales, EQ-5D-5L, and OHIP-14—is pragmatic and appropriate for a large international registry. The absence of a TMJ-specific quality-of-life instrument is acknowledged as a limitation, but this point could be more clearly contextualised.<br><br>It would be useful to briefly justify the complementary roles of OHIP-14 (oral health–specific impact) and EQ-5D-5L (generic health utility), and to clarify whether linguistic and cultural validation of these instruments is ensured across all participating countries. In addition, the authors may wish to state explicitly that the registry could serve as a platform for the future validation or integration of TMJ-specific PROMs as they become available.	We agree with the reviewer, and we have clarified the rationale for the selected PROMs in the revised manuscript. OHIP-14 was chosen to capture oral health–specific impacts that are particularly relevant to TMJ pathology and its functional consequences, while EQ-5D-5L provides a generic measure of health-related quality of life that enables comparisons across conditions and health-economic analyses.<br><br>Both instruments are widely used internationally and have undergone linguistic and cultural validation in multiple languages, which supports their use across participating countries. We have added this clarification to the manuscript.<br><br>We also acknowledge the absence of a TMJ-specific quality-of-life instrument as a limitation and now explicitly state that the registry may serve as a platform for the future integration or validation of TMJ-specific PROMs as they become available, without overburdening participants during this exploratory phase.	*Page 12, line 22 has been modified as follows:*<br><br>restrictions) to 10 (liquids only).<br>The **EQ-5D-5L** is a standardized instrument for assessing health-related QoL and provides a generic measure that enables comparisons across different health conditions as well as health-economic analyses. It includes five dimensions: mobility, self-care, usual activities, pain/discomfort, and anxiety/depression. Each dimension has five levels of severity, ranging from no problems (level 1) to extreme problems (Level 5). The level chosen by the respondent for each dimension creates a 5-digit health profile (38). Validated translations are available in the local languages of all participating study sites.<br><br>The **OHIP-14** was chosen to capture oral health–specific impacts that are particularly relevant to TMJ pathology and its functional consequences. The

			instrument measures oral health-related QoL by focusing on impairment across three functional status dimensions — social, psychological, and physical — which correspond to four of the seven QoL dimensions proposed by Patrick and Bergne<br><br>Validated translations of the OHIP-14 are available in the local languages of all participating study sites<br><br>*Page 17, line 23 has been modified as follows:*<br><br>The registry, however, could serve as a platform for the future integration or validation of TMJ-specific PROMs as they become available, without overburdening participants during this exploratory phase.
4	Heterogeneity across centres<br><br>The protocol appropriately acknowledges variability in surgical techniques, implants, and standards of care across participating centres. Given the central importance of this issue, however, it deserves more explicit methodological consideration.<br><br>Clarification would be welcome regarding <u>how centre effects and</u>	We acknowledge that variability in surgical techniques, implants, and standards of care may influence outcomes, and we have incorporated the following measures to address this:<br><br>**Analytical handling of centre effects:**	*Page 14, line 33 has been modified as follows:*<br><br>Analyses will also be stratified by clinically relevant categories, including treatment patterns. Longitudinal clinical, functional, and PRO data may be analyzed using mixed-effects

	geographic variability are expected to be handled analytically in future analyses (for example, through stratified analyses or multilevel modelling). It would also be helpful to indicate whether minimum data-completeness thresholds are required for centre participation and whether any training or harmonisation procedures are planned for key assessments, such as mandibular movement measurements.	Centre effects and geographic variability will be addressed analytically depending on the distribution of patients across participating centres and treatment regions. Distribution of number of patients per center will be assessed descriptively. If the number of patients per centre is sufficient, centre or treatment region effects will be explicitly considered in the analyses. Centres with very small numbers of patients may be combined at the regional level to ensure adequate sample size and model stability.<br><br>In future analyses, centre effects and geographic variability will be addressed using **multilevel (hierarchical) modelling**, with patients nested within regions, to account for clustering and potential differences in practice patterns.<br><br>For time-to-event outcomes, multivariable Cox regression models for the survival analysis will be performed, considering treatment regions as covariate to account for geographic variability.<br><br>**Data completeness thresholds and harmonization procedures:**	models for repeated measures, allowing inclusion of all available data and minimizing the impact of missing data. Time-to-event analyses are planned, with survival status recorded at each scheduled FU. Kaplan–Meier methods will estimate survival probabilities, and multivariable Cox proportional hazards models will be used where event numbers permit.<br>Center effects and geographic variability will be addressed using multilevel (hierarchical) models with patients nested within regions to account for clustering and differences in practice patterns. For time-to-event outcomes, region (or treating center) will additionally be included as a covariate in Cox regression models when sample sizes permit.<br><br>*Page 15, line 15:*<br>As the registry is embedded within routine clinical care, assessments are performed according to each center's standard practice. To support consistent data capture, all site investigators and study coordinators will receive structured

		As this is an international prospective registry embedded within standard clinical care, participating surgeons are not instructed on how to perform clinical assessments, including mandibular movement measurements, which are conducted according to each centre's routine practice. This approach is intentional to reflect real-world care.<br><br>To ensure consistent and high-quality data capture, all study site Principal Investigators, sub-investigators, and study coordinators undergo a defined training programme prior to enrolling the first patient. Training is provided during the site initiation visit and covers registry procedures, inclusion and exclusion criteria, use of the electronic case report form (eCRF), and general principles of ISO 14155 and Good Clinical Practice (GCP).<br><br>A minimum data-completeness threshold is not defined, but data completeness is monitored centrally through the eCRF system, and sites are supported to address missing or inconsistent entries, while maintaining the pragmatic nature of the registry. We have	training prior to enrollment, covering registry procedures, eligibility criteria, use of the electronic case report form (eCRF), and adherence to Good Clinical Practice principles. Data completeness is monitored centrally through the eCRF system, with sites supported in resolving missing or inconsistent entries.

		clarified this approach in the revised manuscript.	
5	Statistical analysis plan (SAP)<br><br>For a protocol manuscript, the statistical section is appropriate, but it could benefit from greater anticipatory clarity. <u>Explicitly stating that no formal hypothesis testing is planned in initial registry reports would help manage reader expectations.</u> Similarly, it would be useful to clarify that <u>inferential analyses will be considered exploratory unless prespecified in the statistical analysis plan.</u><br><br>Additional clarification on the planned handling of missing data beyond descriptive reporting, and on whether time-to-event analyses (for example, revision surgery) are envisaged, would further strengthen this section.	Please see also our reply to Question 2, and Reviewer 1, questions 4, 6, and 8.<br><br>We have revised the statistical analysis section to clarify that, given the design as an international, multicentre prospective registry, no formal hypothesis testing is planned in the initial registry reports and that all inferential analyses will be considered exploratory unless prespecified in the statistical analysis plan.<br><br>We have also expanded the description of the handling of missing data. Descriptive analyses will be performed using available data, and, depending on the size of the study population, mixed-effects models for repeated measures may be applied for longitudinal clinical, functional, and patient-reported outcomes. These models allow inclusion of all available observations and mitigate the impact of missing outcome values at individual visits.<br><br>Finally, we have clarified that time-to-event analyses are envisaged. Survival status will be recorded at each scheduled follow-up,	*Page 14 line 20 has been modified as follows:*<br><br>Since the objectives of this registry are descriptive and exploratory in nature, no formal hypothesis testing is planned, and all analyses will be exploratory. The planned sample size of 200 patients is based on the estimated number of eligible participants expected to be enrolled during the 2-year recruitment period. Eligible patients who refuse TMJ replacement will be included in the study but excluded from sample size calculations. Depending on the volume and quality of the data collected during the first 2 years, the registry may remain open thereafter, with patient FU of up to 5 years.<br><br>*Page 14, line 33:*<br><br>Analyses will also be stratified by clinically relevant categories, including treatment patterns. Longitudinal clinical, functional, and PRO data may be analyzed using mixed-effects

		and Kaplan–Meier methods will be used to estimate survival probabilities. Where the number of events allows, multivariable Cox proportional hazards regression models will be applied to explore associations between baseline factors and time-to-event outcomes.	models for repeated measures, allowing inclusion of all available data and minimizing the impact of missing data. Time-to-event analyses are planned, with survival status recorded at each scheduled FU. Kaplan–Meier methods will estimate survival probabilities, and multivariable Cox proportional hazards models will be used where event numbers permit.<br>Center effects and geographic variability will be addressed using multilevel (hierarchical) models with patients nested within regions to account for clustering and differences in practice patterns. For time-to-event outcomes, region (or treating center) will additionally be included as a covariate in Cox regression models when sample sizes permit.
6	ensure consistent terminology when referring to TMJ disorders versus TMDs;	Thank you for highlighting this. We have ensured consistent terminology throughout the manuscript when referring to TMJ disorders and TMDs.	The term "TMJ disorder" has now been consistently replaced with "TMD" throughout the manuscript to ensure consistent terminology.

7	correct minor typographical issues (for example, duplicated severity labels within the Helkimo index categories);	Thank you for pointing this out. We have corrected the duplicated severity labels within the Helkimo index categories in the revised manuscript.	*Page 12, line 6 has been modified as follows:*<br>• Patients are classified according to severity as follows:<br>○ D_i 0, clinically symptom free (0 points)<br>○ D_i I, mild dysfunction (1-4 points)<br>○ D_i II, moderate dysfunction (5-9 points)<br>○ D_i III, severe dysfunction (10-13 points)<br>○ D_i III, severe dysfunction (15-17 points)<br>○ D_i III, severe dysfunction (20-25 points)
8	consider including a simple flow diagram illustrating patient pathways (surgery versus refusal).	Thank you for the suggestion. A flow diagram illustrating patient pathways has now been added to the manuscript (Figure 1).	*Figure 1 has now been added to the manuscript*

9	Recommended Literature for the Authors<br>The following references are not mandatory citations, but they could strengthen the conceptual, methodological, and contextual framing of the registry. Each is justified in relation to this protocol.	We thank the reviewer for the suggested literature and for the detailed justification provided. After careful consideration, we have added the first three references in the introduction, as they are directly related to TMDs and align closely with the scope and objectives of the present protocol. The remaining references, while of interest, address topics that fall outside the specific scope and aims of the present manuscript and were therefore not incorporated.	*Page 5, line 1 has been modified as follows:*<br><br>The first-line treatment for TMDs is conservative, with approaches including patient education, physical therapy, pharmacologic management, occlusal appliances, and biofeedback in cases of chronic conditions, often within an interdisciplinary approach addressing physical and psychological factors (2, 13-17).<br><br>*Page 5, line 13:*<br><br>In addition, the readability, reliability, content, and quality of online information TMDs vary widely, which may further contribute to patients' reluctance to pursue surgical treatment (24).
10	Recommended Literature for the Authors**:**<br><br>1. Evaluating online information on temporomandibular disorders: readability, reliability, content and quality – a scoping review protocol<br>BMJ Open, 2025<br>DOI: 10.1136/bmjopen-2025-106870<br>Justification:<br>This protocol addresses the quality and accessibility of TMD-related information from a patient-centred perspective. Its inclusion would reinforce the patient-reported and educational dimension of the present registry, particularly relevant when documenting reasons for surgery refusal and patient decision-making processes. It also aligns methodologically with BMJ Open's emphasis on transparency in protocol reporting.		

	2. Usefulness of biofeedback as a potentially educational treatment of temporomandibular disorders: a systematic review and network meta-analysis<br>BMC Oral Health, 2025<br>DOI: 10.1186/s12903-025-06789-3<br>Justification:<br>This high-level synthesis contextualises conservative and educational interventions within TMD management. Citing this work could help frame TMJ replacement as part of a care continuum, clarifying which patient profiles progress from conservative management to surgical replacement.<br><br>3. Effects of an interdisciplinary approach in the management of temporomandibular disorders: a scoping review<br>International Journal of Environmental Research and Public Health, 2023<br>DOI: 10.3390/ijerph20042777<br>Justification:<br>This review highlights the importance of multidisciplinary care models in TMD. It supports the registry's rationale for collecting broad clinical and psychosocial data, acknowledging that surgical outcomes are influenced by interdisciplinary pathways before and after joint replacement.<br><br>4. Psychological and behavioral factors involved in temporomandibular myalgia and migraine: common but differentiated profiles<br>International Journal of Environmental Research and Public Health, 2023<br>DOI: 10.3390/ijerph20021545<br>Justification:<br>This study reinforces the relevance of psychological and behavioral covariates, which are partially captured in the registry (e.g., anxiety/depression, pain medication use). It provides conceptual support for including psychosocial variables when interpreting surgical outcomes and QoL trajectories.<br><br>5. Diagnostic accuracy of a portable electromyography and electrocardiography device to measure sleep bruxism in a sleep apnea population<br>Clocks & Sleep, 2023<br>DOI: 10.3390/clockssleep5040047<br>Justification:<br>Although not directly focused on TMJ replacement, this study exemplifies objective instrumental assessment of orofacial functional phenomena. It may be cited to support future extensions of the registry toward objective functional or neuromuscular measurements.<br><br>6. Altered pain perception in a young adult with childhood trauma and suspected Riley–Day syndrome: a case report<br>Reports, 2025<br>DOI: 10.3390/reports8020080<br>Justification:<br>This case illustrates atypical pain processing and central sensitization, concepts relevant when interpreting postoperative pain outcomes and variability in patient-reported responses within the registry.<br><br>7. Fourteen-year retrospective cohort study on the impact of climatic factors on chronic

	musculoskeletal pain<br>International Journal of Rheumatic Diseases, 2025<br>DOI: 10.1111/1756-185x.70125<br>Justification:<br>This large cohort study underscores how contextual and environmental modifiers influence chronic pain trajectories. While not specific to TMJ surgery, it supports a broader biopsychosocial interpretation of long-term pain outcomes captured by the registry.<br><br>8. Active Conservative Interventions for Obstructive Sleep Apnea: An Umbrella Review and Meta-Meta-Analysis of Systematic Reviews<br>Journal: Sleep Medicine, 2025<br>DOI: 10.1016/j.sleep.2025.108665<br>Justification:<br>Although this umbrella review focuses on obstructive sleep apnea rather than temporomandibular joint disorders, it provides a high-level methodological example of how heterogeneous clinical populations can be systematically synthesized across multiple conservative and interventional strategies. Of particular relevance to the present registry is its emphasis on stratifying patient responses based on clinical and biological profiles rather than assuming uniform treatment effects.<br>This perspective closely parallels the challenges faced by a global TMJ replacement registry, where patient indications, comorbidities, psychosocial factors, and prior treatments are highly variable. Including this reference could help situate the TMJ registry within a broader precision-medicine and real-world evidence framework, reinforcing the rationale for large, prospective registries as hypothesis-generating platforms rather than purely descriptive datasets.<br><br>9. Instrumental Assessment of Sleep Bruxism: A Systematic Review and Meta-Analysis<br>Journal: Sleep Medicine Reviews, 2024; 74:101906<br>DOI: 10.1016/j.smrv.2024.101906<br>Justification:<br>This systematic review and meta-analysis provides an exemplary methodological framework for the objective, instrument-based assessment of complex biological phenomena characterized by high intra-individual variability and heterogeneous clinical expression. Its in-depth discussion of measurement validity, signal reliability, and phenotype definition is particularly relevant to the present TMJ replacement registry.<br>In the context of this protocol, the paper offers valuable conceptual parallels for interpreting functional outcomes, pain-related measures, and patient-reported data collected across multiple centers with differing clinical practices. Including this reference would strengthen the methodological justification for standardized yet pragmatic outcome assessment within a real-world registry and underscore the importance of distinguishing biologically and clinically meaningful subgroups within apparently homogeneous patient populations undergoing TMJ replacement.<br><br>Overall recommendation to the authors<br>This is a strong, timely, and well-designed protocol with clear potential for long-term impact. The suggested revisions are primarily clarificatory rather than structural and are fully compatible with an ongoing study. Addressing them would enhance transparency, methodological rigor, and the future usability of the registry data.
